# Association of adverse childhood experiences and depression among medical students: the role of family functioning and insomnia

**DOI:** 10.3389/fpsyg.2023.1134631

**Published:** 2023-05-02

**Authors:** Hongxia Tao, Xin Zeng, Mutian Hou, Shanping Chen, Jing Shen, Xiaoyang Liao, Chuan Zou

**Affiliations:** ^1^School of Medical and Life Sciences, Chengdu University of Traditional Chinese Medicine, Chengdu, China; ^2^The Department of General Practice, Chengdu Fifth People’s Hospital, Chengdu University of TCM, Chengdu, China; ^3^Psychological Research and Counseling Center, Southwest Jiaotong University, Chengdu, China; ^4^The Department of Geriatric Medicine, Chengdu Fifth People’s Hospital, Chengdu University of TCM, Chengdu, China; ^5^General Practice Medical Center, West China Hospital, Sichuan University, Chengdu, China

**Keywords:** adverse childhood experiences, family functioning, insomnia, depression, medical students, serial mediation

## Abstract

**Background:**

Few studies have explored the mechanisms linking adverse childhood experiences (ACEs) to depression in medical students. This study aimed to investigate the relationship between ACEs and depression through the serial mediation effect of family functioning and insomnia.

**Methods:**

A cross-sectional survey was conducted with 368 medical students from university in Chengdu in 2021. The participants were asked to complete four self-report questionnaires, including ACEs scale, the family APGAR index, the ISI and PHQ-9. Singe and serial mediation analyses were conducted using structural equation modeling by Mplus 8.3.

**Results:**

ACEs had a significant direct effect on depression (*β* = 0.438, *p* < 0.001) and through three significantly indirect pathways: (1) through family functioning (*β* = 0.026, 95% CI: 0.007–0.060), accounting for 5.9% of the total effect; (2) through insomnia (*β* = 0.103, 95% CI: 0.011–0.187), accounting for 23.5% of the total effect; and (3) through the serial mediators involving in family functioning and insomnia (*β* = 0.038, 95% CI: 0.015–0.078), accounting for 8.7% of the total effect. The total indirect effect was 38.1%.

**Limitations:**

This cross-sectional study prevented us from establishing causality.

**Conclusion:**

This study highlights the role of family functioning and insomnia as serial mediators of the relationship between ACEs and depression. Findings help to elucidate the mechanism that underlines the pathway between ACEs and depression in medical students. These findings may indicate developing measures to strengthen family functioning and improve insomnia aiming to reduce depression in medical students with ACEs.

## 1. Introduction

Depression has been proven to be one of the most common health issues among college students (Ibrahim et al., 2013). Medical students have a higher rate of depression than students from other specialties (Iorga et al., 2018) or the general population (Brenneisen Mayer et al., 2016). In a systematic review, medical students were reported to be at high risk of depression, with a pooled prevalence estimate of 27.2% for depression or depressive symptoms among medical students (Rotenstein et al., 2016). Undoubtedly, medical school is a stressful environment that is highly likely to contribute to the development of depression (Dyrbye et al., 2006; Nasioudis et al., 2015) due to academic pressure, workload, financial hardships, and sleep deprivation (Brenneisen Mayer et al., 2016; Fawzy and Hamed, 2017). Additionally, the Chinese medical education system differs from Western or other Asian countries, with a heavy and demanding medical curriculum predisposed to high mental distress levels (Zeng et al., 2019). Depression can negatively influence medical students, including poor academic performance, school dropout, alcoholism, substance abuse, internet addiction, and suicidal thoughts and attempts (Stewart et al., 1999; Tyssen et al., 2001; Midtgaard et al., 2008; Walkiewicz et al., 2012; Zhang et al., 2018). Nearly one-third of medical students worldwide are affected by depressive symptoms, yet the treatment rate is relatively low (Puthran et al., 2016). So, identifying the factors contributing to depression and gaining a better understanding of how depression in medical students occurs can help prevent and treat it.

### 1.1. Adverse childhood experiences and depression

Adverse childhood experiences (ACEs) are an array of vulnerability factors for the youth. Traditionally, ACEs refer to several negative elements of the developmental process, including family dysfunction, physical/emotional abuse and sexual abuse (Ford et al., 2014). Notably, a recent study has expanded the definition of ACEs to emphasize subsequent adversity occurring outside the family and the socio-context of intra-family adversities (Karatekin and Hill, 2019). Experiencing those adversities is associated with the internal processing of negative events, which will impact future mental and physical health and lead to certain psychosomatic diseases in adolescents (Karatekin and Hill, 2019). These negative outcomes, which are linked to childhood maltreatment represented by ACEs, have a detrimental effect on the development in childhood or even later (Haavet et al., 2004; Polese et al., 2022). Human birth theory can be applied to describe the mechanism from ACEs to personal development, which states that early-life stress, often caused by the defective relationship ACEs are responsible for between infants and main caregivers, may determine mental diseases even in adulthood and threaten the general personal development (Maccari et al., 2017).

Then we focus on the association between ACEs and depression in medical students. Depressive disorders are characterized by cognitive distortions, including negative thinking, dysfunctional personality styles, and dysfunctional attitudes. Negative thinking and distortions in self-perception are hallmarks of depression episodes (Wilkinson and Blackburn, 1981). Various factors contribute to depressive symptoms. Among those, ACEs are considered a strong factor. Indeed, childhood is a period of sensitive brain and psychological development, where both positive and negative experiences can influence maturation, cumulatively across the life course (Ferraro and Shippee, 2009). Children exposed to ACEs may internalize the belief that adverse events are stable and have negative consequences, thus developing a negative cognitive style (Rose and Abramson, 1992). Exposure to harsh parenting styles or maltreatment during childhood increases individuals’ vulnerability to mood disorders (Alloy et al., 2006), possibly permanently altering the stress response system, sensitizing individuals to stress later, and leading to early onset and severe course of the disorder (Post et al., 2001). Stress process theory suggests that adverse experiences test a person’s adaptive abilities (by utilizing resources such as social support) and that the effects multiply over time (Schafer and Ferraro, 2013). As a chronic stressor, ACEs are more likely to predispose victims to future mental illness (Jimenez et al., 2017; Westermair et al., 2018). Studies have shown that ACEs, such as physical abuse, emotional abuse, and sexual abuse, are closely related to depression (Giano et al., 2021; King, 2021). Furthermore, depressed patients with ACEs are more likely to be unresponsive to common treatments (Nanni et al., 2012; Nelson et al., 2017). It is particularly urgent to explore the mechanisms underlying the processes that mediate ACEs and depression to develop psychological intervention strategies that directly target depression with ACEs. Thus, we propose Hypothesis 1:

*Hypothesis 1 (H1):* ACEs can significantly predict depression among medical students.

### 1.2. Family functioning as a mediator

Family functioning is defined as the perception of individuals about the overall function of the family (Suarez Cuba and Espinoza Ma, 2014). Family is where children first observe and learn interpersonal relationships. It is through the family that children learn about behavior, expectations, and interpersonal relationships (Stocker and Youngblade, 1999). Children valued by their parents and given tools to navigate interpersonal relationships are more likely to overcome adversity and build strong social relationships in later life (Agaibi and Wilson, 2005; Lipschitz-Elhawi and Itzhaky, 2005). ACEs may impair family functioning (Schneider et al., 2019). Adversity early in life leads to worsening later subjective well-being (Yang et al., 2022). Individuals who have suffered from ACEs may develop a negative cognitive style (Hartley, 2002), perceiving others as less trustworthy, and this change in trust processing may result in “social thinning” (a decrease in the range and quality of social relationships), increasing the risk of emotional and behavioral self-regulation problems (Neil et al., 2022). According to the deterioration theory of social support (Barrera, 1986; Kaniasty and Norris, 1993), harsh parenting styles and abuse can cause problems in the parent–child relationship (Lackova Rebicova et al., 2020), contributing to lower expectations of family support and less perceived family support.

A study found that family therapy programs act as an effective intervention to reduce ACEs-related harm (Stith et al., 2022). The family system theory holds that the family is a complete system composed of family members, and the network of relationships between family members will affect each other’s emotions, thinking and behavior (Beavers and Hampson, 2000), making individuals particularly vulnerable to mood disorders. In addition, according to the McMaster model of family functioning, families serve as environmental bases for their members’ physical, mental and social development (Epstein et al., 1978). A good family function can provide a stable and safe psychological development environment for family members. In contrast, family dysfunction, lack of family communication and tense parent–child relationship cannot meet the basic psychological needs of family members. It can easily trigger depression and other mental problems for family members (Miller et al., 2000). Several studies have shown a negative correlation between family functioning and depression (Souza et al., 2014; Shao et al., 2020; Wang et al., 2022). Family conflict and family unhappiness play a vital part in this process of family dysfunction. As reported by Polese et al. (2022), family conflict and unhappiness were associated with psychosomatic disorders such as headaches in children. Those psychosomatic disorders impair interpersonal and intrapersonal functions and are detrimental to multiple health outcomes. The decline observed in physical and emotional health conditions, which may worsen because of various stressors medical students experience, is likely to facilitate the development of depression (Berryhill et al., 2018; McKerrow et al., 2020). Therefore, the protective role of family functioning in preventing emotional disorders in medical students cannot be ignored. Consistent with theories and findings, we propose the following:

*Hypothesis 2 (H2):* Family functioning acts as a mediator between ACEs and depression. Specifically, ACEs will be negatively related to family functioning, which will, in turn, make medical students more prone to depression.

### 1.3. Insomnia as a mediator

According to DSM-5, insomnia is defined as the experience of having problems falling asleep, maintaining sleep, or suffering from early morning awakening. Numerous epidemiological studies have discovered an association between ACEs and adult sleep problems (Koskenvuo et al., 2010; Chapman et al., 2011; Greenfield et al., 2011). A large number of retrospective studies have found that adults who have ACEs are more likely to suffer from insomnia than those who do not (Bader et al., 2007b; Zhabenko et al., 2012; Schäfer and Bader, 2013; Kajeepeta et al., 2015). It is well-known that exposure to ACEs can be traumatic, evoking toxic stress responses that have immediate and long-term adverse physiological and psychological impacts. Rumination tends to engage in preservative and non-constructive thoughts and a negative reflection on the problems and feelings in the past or present. A clinical study showed that experienced ACEs significantly predicted a higher level of rumination (Cameron et al., 2018). Rumination about adverse events and self-reflection can disrupt sleep in insomnia disorder (Galbiati et al., 2018). Insomnia could increase the risk and severity of depression (Cunnington et al., 2013; Grandner and Malhotra, 2017; Lang et al., 2017). A meta-analysis showed a combined RR of 2.27 for depression among those with insomnia (Li et al., 2016). Insomnia in medical students may affect their memory and learning ability, leading to worse academic performance (Bahammam et al., 2012; El Hangouche et al., 2018) and further increasing their risk of depression. Furthermore, specific intervention strategies targeting insomnia improved depression (Gebara et al., 2018). Based on the theoretical and empirical evidence presented above, we put forward the following hypothesis:

*Hypothesis 3 (H3):* Insomnia acts as a mediator between ACEs and depression. Specifically, ACEs will be positively related to insomnia, which will, in turn, make medical students more prone to depression.

### 1.4. Family functioning and insomnia as serial mediators

Family functioning, such as family support, is positively associated with adolescent development and is a protective factor (DeVore and Ginsburg, 2005). In the family-centered Chinese culture, family support is probably the most important source of social support for university students (Yang et al., 2021). Positive family functioning improves adolescent sleep quality (Sadeh et al., 2000). Conversely, deficiency in family functioning is strongly associated with increased insomnia symptoms (Bernert et al., 2007). According to emotional security theory, family dysfunction may interfere with children’s feeling of security in their families and negatively impact their adjustment (Cummings and Davies, 1996). Cognitive-affective model of physiological arousal also proposes that adverse family experiences may affect cognitive or emotional self-regulatory processes to stress, which then affect physiological responses (Luecken et al., 2005). Family dysfunction could contribute to abnormal cognitive appraisals of the environment and possibly hypervigilance to threats. Sleep and hypervigilance are physiologically conflicting (Buckley and Schatzberg, 2005), so family dysfunction may lead to insomnia.

Dysfunctional families can fail to adequately promote the proper development of children’s sleep habits due to their inherent disorganization (Billows et al., 2009; Spilsbury et al., 2017; Peltz et al., 2019). In incohesive families, parents often fail to act as role models to model and enforce proper sleep hygiene in children and adolescents who depend more on their parents for learning (Cousins et al., 2007). Adolescents exposed to family conflicts are at greater risk of experiencing insomnia symptoms (Gregory et al., 2006). Family conflict and unhappiness may lead to the deterioration of physical health and the development of headaches, thus affecting sleep quality. Moreover, sleep disturbances were typical depressive symptoms of children with headaches (Polese et al., 2022). Poor sleep and insomnia produced by poor family functioning are believed to partly account for why medical students reported higher levels of depression, anxiety, and burnout than other college graduates and worse emotional health during their training compared to their matriculation (McKerrow et al., 2020). Thus, we expect a serial mediation model and put forward the following hypothesis:

*Hypothesis 4 (H4):* Family functioning and insomnia will sequentially mediate the relationship between ACEs and depression. Specifically, ACEs will be negatively related to family functioning, which might also be negatively related to insomnia, finally making medical students more prone to depression.

### 1.5. The present study

Regrettably, to our knowledge, no studies have examined whether family functioning and insomnia can both simultaneously and sequentially mediate the link between ACEs and depression in medical students. Therefore, the present study sought to bridge the gaps with the conceptual model mentioned. We strive to explore the mechanism in the relationship further, specifically, the mediating roles of family functioning and insomnia. This study aims to provide university regulators and policymakers with a new perspective on improving medical students’ depression by intervening in their family functioning and sleep quality.

**Figure 1:**
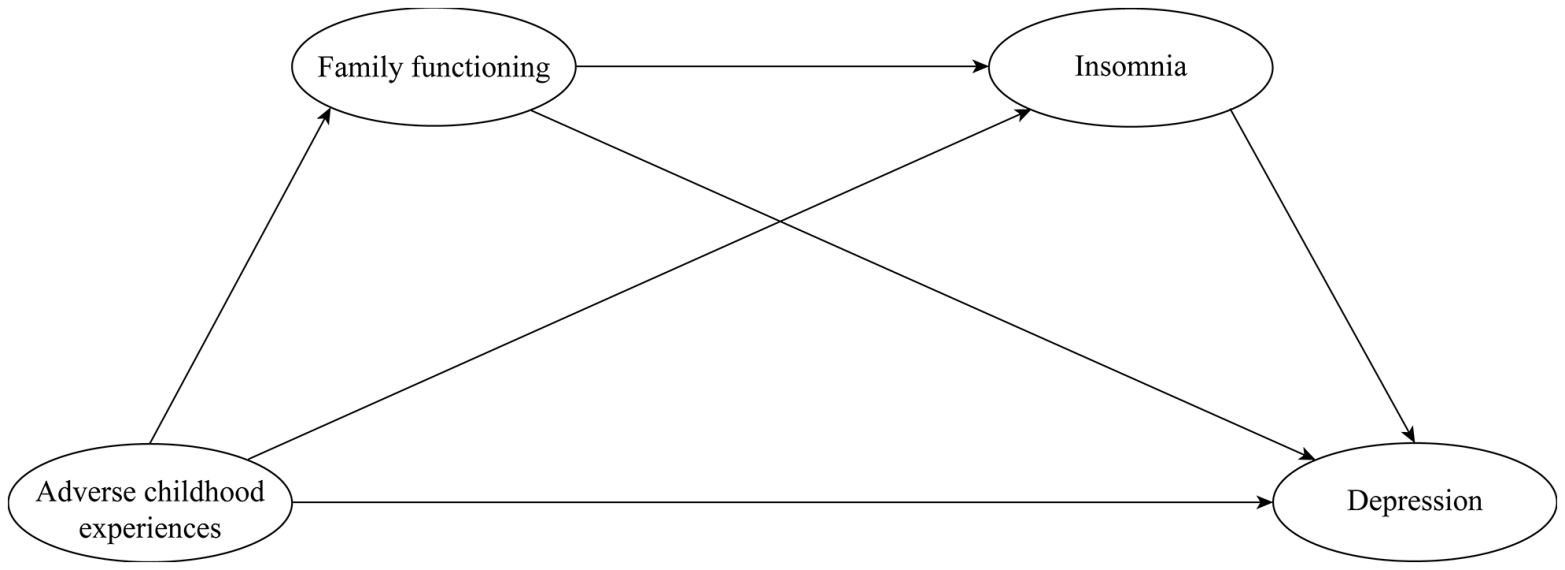
The proposed serial mediation model of the association between adverse childhood experiences (ACEs) and depression.

In addition, previous studies have indicated that having one or more chronic conditions increased the likelihood of presenting with depressive symptoms (Nan et al., 2012; Wang et al., 2015). In people with chronic diseases, adequate family support is considered a protective factor against depressive symptoms, with a stronger association found among women than men (Nan et al., 2012). Another multivariate study showed a strong association between insomnia with gender and chronic diseases (Proserpio et al., 2022). Consequently, we included gender and chronic diseases as control variables. Figure 1 shows the proposed serial mediation model.

## 2. Methods

### 2.1. Participants and procedure

A cross-sectional survey of undergraduate medical students was conducted at the Chengdu University of Traditional Chinese Medicine from October to November 2021. A total of 407 third-year medical students were invited to fill out the questionnaire after class, and 376 questionnaires were returned. We excluded questionnaires with key information omission or logical errors, and eventually obtained 368 valid questionnaires, suggesting an efficiency rate of 90.42%. All participants gave their written informed consent prior to the present study. The research protocol was approved by the institutional review board of the Affiliated Chengdu fifth people’s hospital of Chengdu University of Traditional Chinese Medicine.

### 2.2. Measures

#### 2.2.1. Demographic characteristics

Demographic data included gender, religion, family origins, psychiatric history, parental marriage, chronic diseases, and left-behind experiences. Based on the available literature, left-behind experience is those aged 18 or below who have the experience of continuing to live in their hometown when one or both parents migrate to cities for work for at least 6 months (Gao et al., 2010; Račaitė et al., 2021).

#### 2.2.2. Measurement of adverse childhood experiences (ACE-ASF)

The ACE scale was adapted from the Adverse Childhood Experience of Abuse Short Form (ACE-ASF) which originated from the Romanian Health Behavior in School Children Study (HBSC) (Meinck et al., 2017). It was used to investigate the effect of various adverse events experienced by participants under the age of 18. Participants were asked to answer 8 questions, including: “Did a parent, guardian, or other household member yell, scream or swear at you, insult or humiliate you,” “Did a parent, guardian, or other household member spank, slap, kick, punch or beat you up,” and “Did someone touch or fondle you in a sexual way when you did not want them to.” We dichotomized and coded the responses to these eight items so that each ACEs was coded as 0 (no) or 1 (yes). The scores were then summed to obtain an ACEs score, with a possible range of 0–8. This method of scoring ACEs is consistent with previous studies (Rojo-Wissar et al., 2019; Sheffler et al., 2019; Yang et al., 2020). This scale demonstrated good overall internal consistency. The Cronbach’s α for the total ACE-ASF scale was 0.745.

#### 2.2.3. Measurement of family functioning (APGAR)

The family APGAR index was developed by Smilkstein and had well-established reliability and validity (Smilkstein et al., 1982). This scale evaluates a family member’s perception of family functioning by measuring an individual’s satisfaction with family relationships. It includes five parameters: adaptation (A), partnership (P), growth (G), affection (A), and resolve (R). Three possible answers are allowed (“hardly ever,” “sometimes,” “almost always”), and the score ranges from 0 to 2 points. The points from each item are calculated to obtain the total score. Higher scores indicate better family functioning. A total score of 0–3 suggests severe family dysfunction, 4–6 moderate family dysfunction, and 7–10 good family functioning (Shao et al., 2020). The Cronbach’s α in the present study was 0.878.

#### 2.2.4. Measurement of insomnia (ISI)

Insomnia Severity Index (ISI) was used to evaluate the severity of insomnia during the past 2 weeks for each participant, which included 7 items (Chung et al., 2011). The participants answered questions on a five-point Likert scale ranging from 0 to 4 (0, not at all; 1, mild; 2, moderate; 3, severe; 4, extremely severe). A total score ranged from 0 to 28, with higher scores indicating greater insomnia symptoms (Yu, 2010). The scoring system is defined as follows: 0–7: no insomnia symptoms; 8–14: mild insomnia; 15–21: moderate insomnia; and 22–28: severe insomnia (Bastien et al., 2001; Morin et al., 2011). Assessment of internal consistency in the current study yielded Cronbach’s α of 0.889.

#### 2.2.5. Measurement of depression (PHQ-9)

Depression severity was measured with the Patient Health Questionnaire 9 (PHQ-9), which is a self-report questionnaire designed to explore the depression symptoms experienced by patients during the 2 immediately preceding weeks. The questionnaire included 9 items that are scored on 4-point ranging from 0 to 3 (0, not at all; 1, several days; 2, more than half of all the days; 3, nearly every day). The total score ranged from 0 to 27, with a higher score indicating greater severity of depressive symptoms (Du et al., 2017). Various cut-off scores allow for the determination of different degrees of depression: 0–4: no depressive symptoms; 5–9: mild depressive symptoms; 10–14: moderate depressive symptoms, 15–19: moderate to severe depression; and 20–27: severe depressive symptoms. In our study, a summed score of 10 or above indicated a major depressive disorder (Kroenke et al., 2001). The internal consistency of the PHQ-9 in the present study was very good, with the Cronbach’s α being 0.893.

### 2.3. Statistical analysis

Epidata 3.1 software was used to build the database, and the data were imported into SPSS 25.0 software for demographic descriptive analysis, common method bias tests and correlation analysis after being checked for consistency and correcting errors using double entry. Descriptive statistics were used to calculate the frequency of sample characteristics of the study population, and the results were presented as mean, standard deviation (SD), or percentage (%). The differences between the non-depressed/depressed group in demographic characteristics were analyzed. A chi-square test was used for categorical variables. Pearson correlation analysis was used to analyze the correlations between the four variables. Mplus 8.3 was used to model the structural equation model (SEM). In this study, we used ACEs as independent variables, depression as the outcome variable, family functioning and insomnia as mediators, and gender and chronic diseases as covariates. To assess whether the measurement model and structural model fit the data, we chose the following standard fit indices suggested by previous researchers (Markus, 2012): CFI, TLI, RMSEA, and SRMR. When the CFI and TLI values are above 0.90, and the RMSEA and SRMR values are below 0.08 (Hu and Bentler, 1999), the measurement model and structural model would be considered acceptable. We used the 95% bias-corrected bootstrap confidence interval based on 5,000 bootstrapping to test the mediating effects. According to Mackinnon et al. (2004), when the bootstrap confidence interval (CI) for a mediating effect does not include 0, the mediating effect can be considered significant. Two-sided *p*-values of < 0.05 were considered statistically significant.

## 3. Results

### 3.1. Demographic characteristics

A total of 376 undergraduate medical students completed the questionnaire, and 368 valid questionnaires were obtained. Among them, 166 (34.3%) were male and 202 (65.7%) were female; 356 (96.7%) were Han Chinese and 12 (3.3%) were ethnic minority; 155 (42.1%) were urban and 213 (57.9%) were rural; 37 (10.1%) students had divorced parents. 22 (6.0%) students smoked; 6 (1.6%) consumed alcohol >3 times per week; 20 (5.4%) had a family history of mental disorders; 193 (52.4%) had been left behind in childhood, and 69 (18.8%) had chronic diseases. 141 (38.3%) had experienced mild depression, 46 (12.5%) had experienced moderate depression, 15 (4.1%) had experienced moderate to severe depression and 9 (2.4%) had experienced severe depression. In addition, 172 (46.7%) had suffered at least one ACEs, 167 (45.4%) had suffered at least one physical/emotional abuse and 23 (6.3%) had suffered sexual abuse. The Chi-square test showed that depression was significantly associated with gender and chronic diseases (Table 1).

**Table 1:** Characteristics of participants according to PHQ-9 scores >9 (*N* = 368).

Variable	Total	Non-depressed (*N* = 298), *n* (%)	Depressed (*N* = 70), *n* (%)	*p*-value
*Gender*				0.025
Male	166 (45.1%)	126 (42.3%)	40 (57.1%)	
Female	202 (54.9%)	172 (57.7%)	30 (42.9%)	
*Nationality* ^*a*^				0.706
Han Chinese	356 (96.7%)	289 (97.0%)	67 (95.7%)	
Minority	12 (3.3%)	9 (3.0%)	3 (4.3%)	
*Family origin*				0.683
Urban	155 (42.1%)	124 (41.6%)	31 (44.3%)	
Rural	213 (57.9%)	174 (58.4%)	39 (55.7%)	
*Parents’ marriage*				0.368
Non-divorced	331 (89.9%)	266 (89.3%)	65 (92.9%)	
Divorced	37 (10.1%)	32 (10.7%)	5 (7.1%)	
*Smoking*^*a*^				0.156
No	346 (94.0%)	283 (95.0%)	63 (90.0%)	
Yes	22 (6.0%)	15 (5.0%)	7 (10.0%)	
*Drinking*^*a*^				0.320
No	362 (98.4%)	294 (98.7%)	68 (97.1%)	
Yes	6 (1.6%)	4 (1.3%)	2 (2.9%)	
*Psychiatric history*^*a*^				0.077
No	348 (94.6%)	285 (95.6%)	63 (90.0%)	
Yes	20 (5.4%)	13 (4.4%)	7 (10.0%)	
*Left-behind experience*^*c*^				0.382
No	175 (47.6%)	145 (48.7%)	30 (42.9%)	
Yes	193 (52.4%)	153 (51.3%)	40 (57.1%)	
*Chronic diseases*^*a*^^,^ ^*b*^				0.007
No	299 (81.3%)	250 (83.9%)	49 (70.0%)	
Yes	69 (18.7%)	48 (16.1%)	21 (30.0%)	

ACE-ASF, Adverse Childhood Experiences Questionnaire Abuse Short Form; APGAR, Family APGAR Index; ISI, Insomnia Severity Index; PHQ-9, Patient Health Questionnaire-9.

a Fisher exact test.

b Chronic diseases are defined as diseases of long duration that generally have a slow progression (WHO 2008). The most common chronic diseases among students include chronic gastritis, gastroduodenal ulcers, asthma, anxiety, depression, chronic urticaria, migraine, neurodermatitis, and hyperthyroidism or hypothyroidism.

c Left-behind experience: the experience of continuing to live in their hometown when one or both parents migrate to cities for work for at least 6 months.

### 3.2. Common method bias

In our study, students who participated were ensured that their responses would be anonymous and confidential to control common method bias. Harman's single-factor test extracted five factors with eigenvalues greater than 1. The first factor explained 29.81% of the total variances, which is below the recommended threshold of 50% (Shiau and Luo, 2012). So we could conclude that common method bias is not serious in this study.

### 3.3. Correlation analyses

Means, standard deviations (SD), and Pearson correlations of each study variable were shown in Table 2. ACEs were significantly negatively correlated with family functioning (*r* = −0.204, *p* < 0.01), while was significantly positively correlated with insomnia (*r* = 0.108, *p* < 0.05) and depression (*r* = 0.237, *p* < 0.01). Moreover, family functioning was significantly negatively correlated with insomnia (*r* = −0.320, *p* < 0.01) and depression (*r* = −0.412, *p* < 0.01). Finally, insomnia was significantly positively correlated with depression (*r* = 0.658, *p* < 0.01). The significant correlations between the variables initially supported our hypotheses.

**Table 2:** Means, standard deviations, and correlation of ACEs, family functioning, insomnia and depression (*N* = 368).

Variable	*M* ± SD	1	2	3	4
1. ACEs	1.16 ± 1.58	1			
2. Family functioning	6.29 ± 2.82	−0.204^***^	1		
3. Insomnia	7.23 ± 5.29	0.108^*^	−0.320^***^	1	
4. Depression	6.15 ± 4.95	0.237^***^	−0.412^***^	0.658^***^	1

^*^p < 0.05; ^***^p < 0.001.

### 3.4. Total effect model

Firstly, before analyzing the serial indirect effects model, we established a total effect model to examine the effect of ACEs on depression. Following our correlation results, controlling for gender and chronic diseases, the results of the model revealed a good fit to the data: *χ*^2^*/*df = 3.251, CFI = 0.957, TLI = 0.929, RMSEA = 0.078, SRMR = 0.058. As control variables in the model, gender reported a negative effect on depression (*β* = −0.103, *p* < 0.05), whereas chronic diseases showed a positive effect on depression (*β* = 0.112, *p* < 0.05). The results indicated a significant effect of ACEs on depression (*β* = 0.441, *p* < 0.001). Therefore, hypothesis 1 was supported, that ACEs can positively influence depression.

### 3.5. Indirect effects analysis

**Figure 2:**
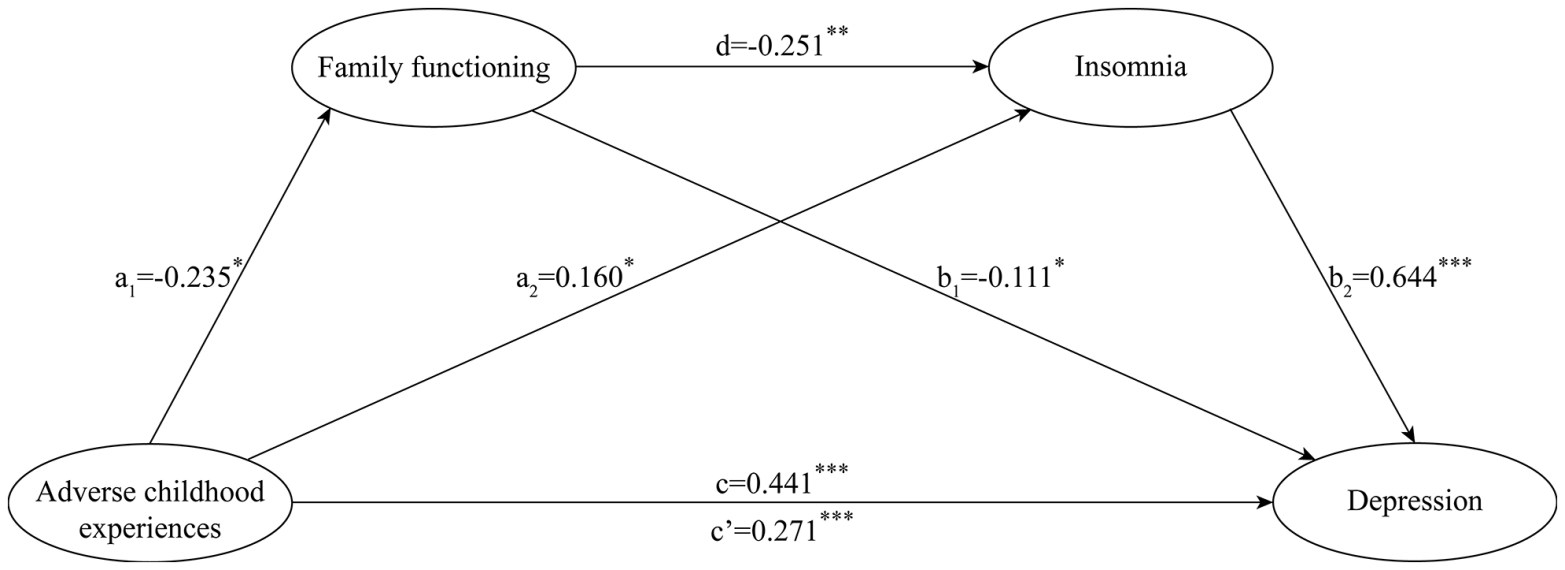
The indirect effects of family functioning and insomnia in the relationship between adverse childhood experiences and depression. ^*^p < 0.05, ^**^p < 0.01, ^***^p < 0.001.

Secondly, controlling for gender and chronic diseases, we constructed a serial indirect effects model (Figure 2) based on theoretical assumptions.

The proposed model shows a goodness of fit to the data: *χ*^2^/df = 3.049, CFI = 0.957, TLI = 0.929, RMSEA = 0.075, SRMR = 0.049. Results of the proposed model (see Figure 2) indicated that the higher ACEs significantly predicted worse family functioning, *β* = −0.235, SE = 0.068, *p* < 0.05, higher insomnia, *β* = 0.160, SE = 0.073, *p* < 0.05, and higher depression, *β* = 0.271, SE = 0.082, *p* < 0.001; the better family functioning significantly predicted lower insomnia, *β* = −0.251, SE = 0.088, *p* < 0.01, and lower depression, *β* = −0.111, SE = 0.051, *p* < 0.05; higher insomnia significantly predicted higher depression, *β* = 0.644, SE = 0.050, *p* < 0.001. Furthermore, as presented in the Table 3, the direct effect of ACEs on depression was significant (*β* = 0.271, *p* < 0.0001, 95% CI = 0.086–0.412), accounting for 61.9% of the total effect. The indirect effect of ACEs on depression through depression was significant (*β* = 0.026, *p* < 0.05, 95% CI = 0.007–0.060), accounting for 5.9% of the total effect; the indirect effect of ACEs on depression through insomnia was significant (*β* = 0.103, *p* < 0.05, 95% CI = 0.011–0.187), accounting for 23.5% of the total effect. Moreover, the indirect effect of ACEs on depression through family functioning and insomnia was significant (*β* = 0.038, *p* < 0.05, 95% CI = 0.015–0.078), accounting for 8.7% of the total effect.

**Table 3:** Bootstrap results for the indirect effects.

Model pathways	Effect	95% CI		Ratio
		Lower	Upper	
ACEs → APGAR → PHQ-9	0.026	0.007	0.060	5.9%
ACEs → ISI → PHQ-9	0.103	0.011	0.187	23.5%
ACEs → APGAR → ISI → PHQ-9	0.038	0.015	0.078	8.7%
Total indirect effect	0.167	0.083	0.260	38.1%
Direct effect	0.271	0.086	0.412	61.9%
Total effect	0.438	0.231	0.594	

ACEs, Adverse Childhood Experiences; APGAR, Family APGAR Index; ISI, Insomnia Severity Index; PHQ-9, Patient Health Questionnaire-9; Ratio, Ratio of the effect to the total effect.

## 4. Discussion

Though Sciolla et al. performed a preliminary analysis to untangle the relationship between ACEs and mental health outcomes among medical students, including depression, the current study attempted to include family functioning and insomnia as mediators to describe the relationship. Our findings showed that depression was positively associated with the history of ACEs in the medical student population. Family functioning and insomnia independently mediated the path from ACEs to depression. Furthermore, family functioning and insomnia played a serial mediating role in the relationship between ACEs and depression.

### 4.1. ACEs and depression

The findings support our Hypothesis 1 that ACEs would positively predict depression among medical students, suggesting that those medical students who report a history of ACEs are at a higher risk of developing depression. Our results align with the study of Ward et al., which demonstrated that ACEs had a substantial impact on mental health outcomes, and septically correlated with an increase in depression in a community sample (Hughes et al., 2017; Ward et al., 2020). Previous study found that childhood emotional abuse, as an aspect of ACEs, is a more potent risk factor for depression than other types of childhood adversity, consistent with cognitive theoretical frameworks positing that individuals may internalize depressive cognitions from abusers that contribute to the development of cognitive attributions most consistent with depression. The other explanation may be continued stress. Early adversity plays a role in the development of depression due to its relationship with adolescent stress burden, viewed as leading contributor to depression. Continued exposure to stress during childhood and adolescence may increase or maintain symptom levels, eventually developing into full syndromal depression. Our study suggested that it is vital to evaluate ACEs when dealing with depression among medical students.

### 4.2. Mediating effect of family functioning

We tested and confirmed that family functioning mediated the relationship between ACEs and depression among medical students, supporting Hypothesis 2. To our knowledge, there is little previous literature on the mediating role of family functioning in the association between ACEs and depression. Our research expands the field of research and fills a gap in this area. Our results align with previous empirical studies, which showed that exposure to ACEs can lead to impaired family functioning (Schneider et al., 2019). The underlying mechanisms of this relationship are not hard to understand. During childhood, parents give their children important feedback about the causes, consequences, and meaning of adverse events internalized by the child into a cognitive style for adverse events (Poletti et al., 2014). Growing up in a family environment characterized by harsh parenting seems to lead to the development of dysfunctional cognitive processing, which increases the risk of perceiving worse family functioning. Garmezy’s theory states that positive relationships within and outside the family are predictors of resilience (Garmezy, 1992). From this perspective, positive aspects of family functioning, such as providing emotional warmth and social control, may contribute to resilience and reduce the risk of adverse psychiatric effects after experiencing adversity (Bradley et al., 2013). Providing emotional warmth and social control are two essential functions of a good family (Chng et al., 2015). The emotional warmth provided by family members, from which children receive support and protection, reduces the negative emotions caused by ACEs. Therefore, medical students who live in well-functioning families may be at less risk of suffering from depression (Nam et al., 2016). In the literature on depression, family functioning is a protective factor that has been found to reduce the likelihood of experiencing depression (Guerrero-Muñoz et al., 2021; Wang et al., 2021).

### 4.3. Mediating effect of insomnia

In the present study, insomnia was also found to mediate the relationship between ACEs and depression among medical students, supporting Hypothesis 3. Adults with ACEs are inclined to have sleep problems due to increased sleep onset latency, awakenings, motor arousals, body movements, the proportion of sleep time spent on movement and the proportion of sleep time spent on rapid eye movement (REM) sleep, as well as lower sleep efficiency compared to those without ACEs (Bader et al., 2007a,b; Heitkemper et al., 2011; Bader et al., 2013). Moreover, several studies have found that persistent insomnia caused by negative experience is more likely to elevate depression among medical students (Ramón-Arbués et al., 2020; Marelli et al., 2021). Insomnia may increase the risk of depression through several possible mechanisms. On the one hand, insomnia triggers cognitive and emotional changes and disrupts emotional regulation and stability, further contributing to depression (Novati et al., 2008; Jackson et al., 2014). On the other hand, sleep disorders can lead to reduced immunity, which lowers the ability to fight inflammation and infection. Increased inflammatory markers, such as C-reactive protein and interleukin-6, and persistent inflammatory response can form a state of chronic tissue injury, easily resulting in long-term depression (Toda et al., 2019). Besides, as a chronic stressor, insomnia can facilitate chronic activation of the hypothalamic–pituitary–adrenal axis, which is considered to increase the risk of depression (Ehnvall et al., 2004; Balbo et al., 2010; Fernandez-Mendoza and Vgontzas, 2013).

### 4.4. Serial mediating effect of family functioning and insomnia

We found that ACEs influenced depression among medical students through the serial mediation impacts of family functioning and insomnia, supporting Hypothesis 4. Basic psychological needs theory and the organism-environment interaction model suggest that the dynamic interactions between internal and external environmental factors influence an individual’s mental health (Ma et al., 2020). Based on this model, the serial mediation model reveals a crucial potential mechanism that family and lifestyle factors, including family functioning and insomnia involved in the impact of ACEs on depression. Family functioning leads to insomnia may through multiple mechanisms. First, family dysfunction may affect physiologic functioning (Dopp et al., 2000), and physiologic changes may interfere with sleep (Phillips and Mannino, 2005). Furthermore, feelings of insecurity surrounding family dysfunction may result in hypervigilance (Cummings and Davies, 2002), which may lead to insomnia since sleep is incompatible with vigilance (as defined by awareness and responsiveness to the environment) (Dahl, 1996). In addition, worry and rumination have been shown to contribute to insomnia (Espie, 2002; Harvey, 2002), and individuals in dysfunctional families are especially susceptible to these cognitive styles. Finally, family functioning chaos may impair sleep hygiene, which refers to the conditions and practices that promote restful sleep (Gregory et al., 2005). Indeed, a longitudinal association between family dysfunction and insomnia could occur if children develop unsuitable sleep patterns that persist into later life, or live in dysfunctional families when they become adults. We affirm the serial mediating role and highlight the vital role of external (family functioning) and internal (insomnia) factors in influencing depression in medical students. Our findings suggest that consistent with previous research findings, improving family functioning can help alleviate insomnia (Lv et al., 2022). Nevertheless, it is noteworthy to mention that insomnia is regarded as a depressive symptom rather than a distinct mental disorder according to some nosographic arguments, as there is evidence that 41% of depressive patients report sufficient insomnia symptoms to meet the criteria of DSM-IV diagnosis of insomnia which worked as a prodromal symptom of depressive recurrence (Perlis et al., 1997; Stewart et al., 2006).

## 5. Limitations and future directions

Although the study process and statistical analysis were relatively rigorous, and the findings may have some implications for medical students’ mental health, some limitations also require special attention. First, the causality could not be established because of the cross-sectional design. Longitudinal studies are needed to confirm the findings of this study. Second, the questionnaire used in our study to evaluate medical students’ ACEs, family functioning, insomnia and depression were all self-reported. In the future, a combination of self-rating scales and clinically objective diagnosis can be used for mental health evaluation. Third, participants were only from one medical university, and non-random sampling may affect the representativeness and reliability of the findings. Finally, there are potential overlapping components between ACEs and family functioning due to many ACEs coming from family abuse. Although ACEs occur in early life and family functioning is a recent measure for medical students, family functioning is still likely to influence ACEs, considering that family functioning is probably a stable factor. Therefore, researchers are encouraged to investigate the possible bi-directional relationship between ACEs and family functioning by establishing a cross-lagged panel model in the future.

## 6. Conclusion

To summarize, the present study found that ACEs may make medical students vulnerable to depression. Furthermore, family functioning and insomnia serve both parallel and sequential mediating roles in the relationship between ACEs and depression. Our results call for more support and attention to be paid to depression among Chinese medical students. To protect medical students with ACEs from developing depression, university administrators and health policymakers could achieve this by incorporating prevention and intervention programs targeting family functioning and insomnia. The results of this study are also instructive for mental health education in medical universities.

## Data availability statement

The raw data supporting the conclusions of this article will be made available by the authors, without undue reservation.

## Ethics statement

The studies involving human participants were reviewed and approved by the research protocol was approved by the institutional review board of the Affiliated Chengdu fifth people’s Hospital of Chengdu University of Traditional Chinese Medicine. The patients/participants provided their written informed consent to participate in this study.

## Author contributions

CZ and XL conceived and designed the study. HT and XZ collected the data and drafted the manuscript. HT and MH conducted the statistical analyses. JS, XL, and SC advised the data analysis process. MH, CZ, and JS provided critical feedback towards developing the research question and results interpretation and had been involved in manuscript redrafting. All authors contributed to the article and approved the submitted version.

## Funding

This research was supported by the educational reform project of Chengdu University of TCM (JGJD202021), the scientific research project of Chengdu health and family planning commission (no. 2021062), and the scientific research project of Sichuan medical association (no. Q20100).

## Conflict of interest

The authors declare that the research was conducted in the absence of any commercial or financial relationships that could be construed as a potential conflict of interest.

## Publisher’s note

All claims expressed in this article are solely those of the authors and do not necessarily represent those of their affiliated organizations, or those of the publisher, the editors and the reviewers. Any product that may be evaluated in this article, or claim that may be made by its manufacturer, is not guaranteed or endorsed by the publisher.
