# Supplementary material for: Association of adverse childhood experiences and depression among medical students: the role of family functioning and insomnia
Source: Front Psychol. 2023 May 2;14:1134631. doi: 10.3389/fpsyg.2023.1134631 (PMC10185847; doi:10.3389/fpsyg.2023.1134631)
Supplement: Supplementary file 1 [file Table_1.docx]

| **Supplemental Table 1.** Standardized results of the ACE-ASF 2-factor model Confirmatory Factor Analysis with standardized factor loadings (n = 368). | | | | |
| --- | --- | --- | --- | --- |
| Abuse constructs Item: | Physical/Emotional Abuse | | Sexual Abuse | |
|  | *β* | SE | *β* | SE |
| ‘Did a parent, guardian or other household member yell, scream or swear at you, insult or humiliate you?’ | 0.739 | 0.041 |  |  |
| ‘Did a parent, guardian or other household member threaten to, or actually, abandon you or throw you out of the house?’ | 0.624 | 0.050 |  |  |
| ‘Did a parent, guardian or other household member spank, slap, kick, punch or beat you up?’ | 0.740 | 0.042 |  |  |
| ‘Did a parent, guardian or other household member hit or cut you with an object, such as a stick (or cane), bottle, club, knife, whip etc.?’ | 0.690 | 0.046 |  |  |
| ‘Did someone touch or fondle you in a sexual way when you did not want them to?’ |  |  | 0.624 | 0.108 |
| ‘Did someone make you touch their body in a sexual way when you did not want them to?’ |  |  | 0.683 | 0.139 |
| ‘Did someone attempt oral, anal, or vaginal intercourse with you when you did not want them to?’ |  |  | 0.844 | 0.076 |
| ‘Did someone actually have oral, anal, or vaginal intercourse with you when you did not want them to?’ |  |  | 0.808 | 0.111 |
